# The relationship between bullying, learning disorders and psychiatric comorbidity

**DOI:** 10.1186/s12888-023-04603-4

**Published:** 2023-02-21

**Authors:** Lior Weinreich, Stefan Haberstroh, Gerd Schulte-Körne, Kristina Moll

**Affiliations:** Department of Child and Adolescent Psychiatry, Psychosomatics and Psychotherapy, University Hospital, LMU Munich, Nussbaumstrasse 5a, 80336 Munich, Germany

**Keywords:** Childhood bullying, Learning disorders, Psychiatric comorbidity, Path modeling

## Abstract

**Background:**

Both learning disorders and bullying are major sources of public concern. Children with learning disorders often suffer from social rejection, potentially rendering them more susceptible to bullying involvement. Bullying involvement leads to a higher risk towards developing various problems including self-harm and suicidality. Past research on whether learning disorders are childhood bullying risk factors yielded inconsistent results.

**Methods:**

The current study used path analyses on a representative sample of 2,925 German 3rd and 4th grades to examine whether learning disorders are a direct bullying risk factor, or whether their impact depends on psychiatric comorbidity. More so, the current study sought to examine whether associations differ between children with and without learning disorders, compare different bullying roles (i.e., only victim, only bully, or bully-victim), compare gender, and control for IQ and socioeconomic status.

**Results:**

Results indicated that learning disorders are not a direct but rather an indirect childhood risk factor for bully-victim involvement, depending on psychiatric comorbidity with internalizing or externalizing disorders. Regarding the comparison between the samples of children with and without learning disorders, an overall difference and a difference in the path between spelling and externalizing disorders emerged. No difference for different bullying roles (i.e., only victim, only bully) emerged. Negligible differences emerged when IQ and socioeconomic status were controlled. An overall gender difference emerged, compatible with past research, indicating higher bullying involvement among boys compared to girls.

**Conclusion:**

Children with learning disorders are at a higher risk of having psychiatric comorbidity, which in turn renders them at a higher risk of bullying involvement. Implications for bullying interventions and school professionals are deduced.

## Purpose

The main purpose of the current study was to find out whether children with learning disorders are more susceptible to bullying involvement, or if such susceptibility depends on psychiatric comorbidity. Thus, shedding light on the inconclusive nature of the results of previous studies done in this realm. Additional purposes included the comparison between children with and without learning disorders, different bullying roles (i.e., only victim, only bully, bully-victim), gender, and controlling for IQ and socioeconomic status.

## Background

Learning disorders (LD) can be broadly defined as persisting poor academic skills and outcomes according to The Diagnostic and Statistical Manual of Mental Disorders, Fifth Edition (DSM-5; 1). LD symptoms include deficits in: reading (i.e., accuracy, fluency and/or reading comprehension); spelling; and basic math skills such as calculation and fact retrieval [1]. Notably, these difficulties are not accounted by a lack of motivation or access to education, intellectual disabilities, poor vision or poor hearing [1, 2]. LD develop due to both environmental and genetic factors [3]. Heritability rates range between 50 and 70% for reading disorder (i.e., dyslexia), and between 40 and 60% for math disorder (i.e., dyscalculia; 3, 4, 5).

Notably, LD are a major source of public concern worldwide [6]. Alarmingly, up to 40% of North American children read below grade level [7], and 5–15% of children worldwide fulfill diagnostic criteria for LD [1, 2]. Specifically, roughly 4–17% of children fulfill diagnostic criteria for reading disorder; 5–7% for spelling disorder; and 2–13% for math disorder [7–11].

Additionally, children with LD are prone to psychiatric comorbidity [12]. Angold et al.[13] define psychiatric comorbidity as the co-occurrence of two or more distinguished disorders. Two types of comorbidities are distinguished: those belonging to the same diagnostic grouping (i.e., homotypic comorbidity); or to different diagnostic grouping (i.e., heterotypic comorbidity). Correspondingly, LD may co-occur both with one another and with other disorders [1].

Remarkably, comorbidity rates for LD are not only high for homotypic comorbidity (e.g., ranging between 17% and 70% for a comorbid reading and math disorder; 11) but also for disorders from very different diagnostic categories, such as between reading disorder and attention-deficit/hyperactivity disorder (i.e., ADHD; 14, 15). For example, Visser et al. [15] found comorbidity rates of 21% for LD and anxiety disorder; 28% for LD and depression; 28% for LD and ADHD; and 22% for LD and conduct disorder.

Furthermore, children with LD (with or without co-occurring disorders) are prone not merely to academic hardship, but also to truancy, early dropout and social hardship [16, 17]. For example, roughly 25–30% of children with LD suffer from social rejection, compared to 8–16% of children without LD [18]. In turn, this rejection renders children with LD less socially protected, and thus more susceptible to being victims of bullying acts [17].

Bullying is defined as imposed aggressive acts, inflicted by aggressors towards victims, under a power imbalance [19]. Gladden et al. [19] distinguish between two *modes* of bullying: direct – happens in the presence of the victim (e.g., pushing); and indirect – happens in the absence of the victim (e.g., rumor spreading). Furthermore, they distinguish between four *types* of bullying: physical – via physical force (e.g., kicking); verbal – via oral or written discourse (e.g., taunting); relational – via impairment of one’s reputation and social contact (e.g., excluding); damage to property – via stealing or vandalizing (e.g., trashing). Thus, bullying can be inflicted in many forms, negatively impacting everyone involved (i.e., victims of bullying acts, bullies, and bystanders; 20).

Notably, both worldwide and in Germany, bullying is a major source of public concern [21]. About 16% of German students, amongst them boys more so than girls, have been involved in bullying [21]. These estimates are even higher, reaching up to 25%, among German children and young adults undergoing psychotherapy [22]. This is alarming, as both being a victim of bullying and being a bully are associated with behavioral and emotional problems.

*Victims of bullying acts* are characterized by impaired relations with peers, teachers and parents [23]. Additionally, as victimization increases, they become more prone to pessimism, depressive symptoms, lower popularity, somatic complaints, anxiety, self-blame and murderous ideation and behaviors [23–25]. Moreover, victims are at a higher risk towards developing: psychosomatic disorders (e.g., migraines; 26), internalizing disorders (e.g., depression; 27), educational impairments (e.g., test underperformance; 19), psychosis in late adolescence [28], and a variety of long-term problems persisting throughout adulthood, such as self-harm and suicidality [29–32]. Interviews also reveal that victims constantly feel fearful of being bullied again, insecure, isolated, and angry [33].

Parallelly, as victims, *bullies* are characterized by impaired relations with peers, teachers and parents [23]. Additionally, as bullying involvement increases, they become more prone to pessimism, depressive symptoms and murderous ideation and behaviors [23, 25]. Moreover, bullies are also at a higher risk towards developing psychosomatic disorders [26], and psychosis in late adolescence [28]. However, unlike victims, bullies often enjoy popularity and friendships [23, 24].

Previous research on victims of bullying acts revealed the following prominent childhood bullying risk factors: intelligence (e.g., low IQ), high body mass index (i.e., BMI; e.g., obesity), internalizing and externalizing disorders (e.g., anxiety and ADHD, respectively), physical disabilities, socioeconomic status (i.e., SES; e.g., low income), low maternal support, identification as lesbian, gay, bisexual, transgender and related communities (i.e., LGBTQ+), immigrational background, minority religious affiliations, and intersectionality [27, 34–38].

Nonetheless, previous research on other childhood bullying risk factors, and namely having LD, has yielded inconsistent results. Some studies reported an association between LD and victimization [39–42]. Others found that LD was only related to victimization when associated with comorbid disorders, such as ADHD [43], or that LD was unrelated to victimization, but rather other controlled factors such as prior history of victimization emerged as risk factors [44]. Similarly, whereas some studies reported an association between LD and bullying perpetration [45], others found that LD was unrelated to bullying perpetration, but rather other controlled factors such as gender emerged as risk factors [46].

There are several potential explanations for these inconsistent findings:

One possible reason is that previous research investigating LD as a childhood bullying risk factor did not always take psychiatric comorbidity into account. The few existing studies found that children with LD and psychiatric comorbidity (e.g., LD and ADHD) are at a higher risk of victimization [42], and of being bullies and bully-victims [47, 48]. However, these studies did not examine all bullying roles (i.e., only victim, only bully, or bully-victim), or did not examine the effect of difficulties in different learning domains (i.e., reading, spelling, and math). Controlling for psychiatric comorbidity is important as it often co-occurs with LD [11] and as some disorders (e.g., anxiety) have been shown to be childhood bullying risk factors [29].

A second possible reason is that the research was based on very different samples, namely clinical samples, typically developing children, and representative samples, covering the whole distribution of learning skills. For example, Klomek et al. [43] used a sample including only children in general education, whereas Blake et al. [44] also included children in special education. It is possible that the predictive patterns might differ among children with LD, belonging to the lower end of the learning distribution, compared to typically developing children or a sample representing the whole distribution.

A third possible reason is that previous studies did not always take both aspects of bullying into account, namely bullying and victimization. For example, Blake et al. [44] measured only if LD is associated with being a victim, but not with being a bully. Nevertheless, some children, and those with ADHD in particular, are prone to be both bullies and victims (i.e., bully-victims; 17, 37). More so, ADHD related behavior, such as hyperactivity, along with social difficulties, are linked to both conduct disorder and lower popularity [49, 50]. Lower popularity among children with ADHD, in turn, leads to higher rates of victimization [37]. Such victimization may result in being a bully as a form of resistance, in which the child bullies not proactively but rather as a backlash [51]. This bully-victim duality can be explained by the term “negative feedback loop” coined in this context by Simmons and Antshel [52]. This negative loop puts bully-victims at a higher risk towards developing both internalizing and externalizing co-occurring disorders [37, 51, 53].

Finally, the inconsistent results might be a consequence of not taking other potentially relevant factors into account. Notable factors that could affect the association between LD and bullying are gender, IQ, and socio-economic status (SES). The few existing studies on LD as a bullying risk factor that did control for gender, yielded inconsistent results. While Klomek et al. [43], Rose et al. [46] and Turunen et al. [40] found higher bullying involvement rates for boys compared to girls, Blake et al. [44] found no gender differences. Controlling for gender is important as it could influence bullying involvement overall and involvement in specific bullying roles. For example, there is some evidence that girls are more likely to be victims [46]. With respect to IQ and SES, both have emerged as prominent bullying risk factors in previous research (e.g., 34), but the association between LD and bullying and the role of IQ and SES in explaining this relationship has yet to be determined.

The current study sets to investigate the role of LD and psychiatric comorbidity as childhood bullying risk factors in a representative sample of 2,925 German 3rd and 4th graders. Moreover, the study addresses the possible reasons for the inconsistent findings from previous research reviewed above by: Firstly, taking into account co-occurring difficulties, namely both internalizing (i.e., anxiety and depression) and externalizing disorders (i.e., ADHD and conduct disorder); Secondly, analyzing both a representative sample as well as comparing children with and without LD; Thirdly, taking bullying role duality into account (i.e., being both a bully and/or a victim); and fourthly, taking gender, IQ and SES into account. That is, a model was built with learning skills (i.e., reading, spelling and math skills) as exogenous variables, IQ, SES, internalizing and externalizing disorders as the endogenous variables and bully-victim involvement as the outcome variable.

### Aim

The methods employed in the current study aim to answer the following research questions: [1] Is there a direct link between LD/learning skills and childhood bullying, or does the association depend on other co-occurring psychiatric disorders? [2] Do the associations differ when comparing children with and without LD? [3] Do these associations differ when examining either being both a bully and a victim compared to only being a victim or a bully? [4] Do these associations differ for boys compared to girls, and do these associations differ when taking IQ and SES into account?

## Methods

### Participants

Recruitment for the current study targeted families with children in 3rd and 4th grade residing in two different federal states in Germany: Hesse and Bavaria. In Hesse, families were invited via the Ministry of Education and Cultural Affairs (*N =* 25,000), and in Bavaria, families were invited via local registration offices (*N =* 27,734). Overall, 52,734 randomly chosen families were invited and, among them, 4542 families agreed to participate. These recruitment invitations were coordinated by two collaborating institutions from the above mentioned German federal states, respectively: The Leibniz Institute for Research and Information in Education (DIPF) in Frankfurt; and The Clinic for Children and Adolescent Psychiatry, Psychosomatics and Psychotherapy (KJP) in Munich.

This initial sample size (N = 4542) was reduced due to the following applied exclusion criteria: participants did not complete all test items or questionnaires; parents reported in an open-ended question that their child had either a neurological disease, a hearing or a visual problem, or a chromosomal defect; children had an IQ below or equal to 70. Furthermore, to avoid statistical dependence, data for one sibling per sibling-pair was excluded randomly. The resulting final sample size was *N* = 2,925, with a mean age of 9.72 years (*SD* = 7.19 months; range 8.08–11.67), and was constituted of: 52% (*n =* 1520) boys; 48% (*n =* 1405) girls; 47.5% (*n =* 1390) 3rd graders; 52.5% (*n =* 1535) 4th graders.

Among the 2,952 participants of the final sample, 13% (*n* = 373) had LD. Diagnostic criteria for LD (i.e., reading, spelling and/or math disorder) were based on the German clinical diagnostic guidelines[54, 55] and in accordance with the recommendation of the DSM-5 [1]. In order to receive a diagnosis of LD, performance had to be at least 1.5 standard deviations (SD) below the sample’s grade-specific mean in at least one of the different standardized academic tests (assessments detailed below under “Children’s assessments”). Notably, the guidelines also recommend a less stringent criterion of -1 SD, but only when other information supporting the existence of LD is available (e.g., clinical assessments), which was not the case in the current study.

### Data collection

Data for the current study was collected by the collaborating institutions as part of a larger study that explored children’s comorbid LD, whilst taking into account numerous familial and environmental factors. Specifically, data were collected via a web-based application (i.e., app) assessing children’s academic skills and psychopathological profile. The app was developed by a German software company (i.e., Meister Cody), and was downloaded and installed by all invited families using a login code. After logging in, parents were asked to give informed consent, in accordance with the Declaration of Helsinki. Priorly, approval was obtained from the ethics committees in both collaborating institutions.

Participants were instructed to install the app on either a smartphone or tablet within eight weeks after receiving the invitation to take part in the study, and complete various tests and questionnaires. Children had to complete the following tests and questionnaires: academic tests assessing reading, spelling and math skills; a test measuring nonverbal cognitive abilities; four questionnaires assessing their psychopathological profile; and a questionnaire about bullying involvement. These had to be completed in four separate days (i.e., each day composed a session). An additional fifth session, in which children were asked to complete a piloted spelling test was optional and not part of the current analysis. Each session lasted roughly 30–45 min. To avoid tediousness and encourage engagement, sessions were gamified and embedded in a story about a magician. Parents had to complete various questionnaires about their children in the course of one session. For the current study, the explored data obtained from participating children was composed of academic tests, a test measuring nonverbal cognitive abilities (IQ), and a bullying questionnaire. Parallelly, the explored data obtained from participating parents was composed of a psychopathological profile questionnaire, and a familial and environmental factors questionnaire.

### Measures

#### Children’s assessments

Reading skills were assessed using the Würzburger silent reading test – revised (WLLP-R; 56). Retest-reliability obtained from the test manual is: r _tt_ = 0.82 for 3rd graders, and r _tt_ = 0.80 for 4th graders. The test is designed to assess word reading fluency. It is composed of 180 items, and is suitable for children from 1st -4th grade. Each item is composed of a word and four images. Children had to read the word and identify the image that matches the word as fast as possible. In the course of five allocated minutes, children had to match as many items as possible. The relevant score is the number of items processed correctly within the time limit (max. score = 180).

Spelling skills were assessed using the long version of the Weingartener spelling test for basic vocabulary for 3rd graders (WRT 3+; 57) and 4th graders (WRT 4+; 58). Retest-reliability obtained from the test manual is: r _tt_ > 0.92 for 3rd graders, and r _tt_ > 0.93 for 4th graders. The test is designed to assess spelling accuracy. The WRT 3 + is composed of 55 items, and the WRT 4 + of 60 items. During the test, children had to fill in dictated words that were presented in a sentence frame. The relevant score is the number of correctly spelled words (max. score = 55 and 60 for 3rd and 4th grade, respectively).

Math skills were assessed using the arithmetic scale of the Cody math test (CODY-M 2–4; 59). Retest-reliability obtained from the test manual of the arithmetic scale is r _tt_ = 0.85. The arithmetic scale is composed of four subtests: addition (7 items), subtraction (7 items), multiplication (4 items), and place holder (4 items). The test is suitable for children from 2nd -4th grade. During the test, children were presented with audible instructions and instructed to solve the written questions by typing in the correct answer (e.g., 57 − 23 =__). The relevant score is the number of correct answers (max. score = 22).

Nonverbal cognitive abilities (i.e., IQ) were assessed using the Culture Fair Intelligence Tests (CFT 20-R; 60). Retest-reliability obtained from the printed test manual is: r _tt_ > 0.80. The test is suitable for children from ages 8.5-19.11. For the current study, three of the four subtests that were compatible for online usage (i.e., sequences of drawing, classifications and matrices) were used, each composed of 15 items. During the test, children were presented with tasks varying in complexity, and instructed to recognize figural relationships and solve logical problems within a time limit (4, 4 and 3 min, respectively for the above-mentioned subtests). The relevant score is the number of correct answers (max. score = 45).

Bullying involvement was assessed using the short German version of the revised Olweus bully/victim questionnaire (OBQ; 61, 62). The questionnaire’s reliability measure obtained from the test manual is: Cronbach’s alpha = 0.84. The test is designed to assess the frequency of bullying involvement both as a bully and as a victim (e.g., “I called a classmate an ugly name”, “I have been made fun of and teased in a mean way”). The test is composed of 18 items: 9 assessing bullying perpetration; and 9 assessing victimization. Two items were excluded because of their high complexity level (i.e., “I called a classmate an ugly name because of the color of their skin or where they came from”, and “I was called an ugly name because of the color of my skin or where I came from”). Thus, the final test was composed of 16 items: 8 assessing bullying perpetration and 8 assessing victimization. Since two of the options on the five-point Likert scale were too similar for the 3rd and 4th graders that participated (i.e., “two or three times a month” vs. “once a week”), they were merged. Thus, items were scored on a four-point Likert scale (scores ranged between 0 and 3). The relevant scores are the z-standardized summed scores for bullying perpetration (based on the 8 bullying perpetration items), victimization (based on the 8 victimization items), and bully-victim involvement (based on all 16 items). In order to clearly differentiate between the different roles, z-scores larger than 1 (SD > 1) indicated either bullying perpetration, victimization or bully-victim involvement, and scores equal or lower than 0 (i.e., the mean) indicated either no bullying perpetration, no victimization or no bully-victim involvement.

#### Parents’ assessments

Psychopathological profiles were assessed using three scales from a standardized parental questionnaire, the diagnostic system for mental disorders according to ICD-10 and DSM-IV, for children and adolescents (DISYPS-II; 63). The three scales assess children’s symptoms of depression, conduct disorder, and ADHD. The reliability measures obtained from the test manual are: Cronbach’s alpha = 0.89, 0.89 and 0.94, respectively for the above-mentioned scales. The three scales comprise 87 items: 42 items about depression; 25 items about conduct disorder, which included nine items about oppositional-aggressive behavior and 16 about antisocial-aggressive behavior; and 20 items about ADHD, which included nine items about inattention, seven about hyperactivity, and four about impulsivity. Items were scored on a four-point Likert scale (scores ranged between 0 and 3). The raw score (= summed score) of each scale is transferred to a standardized score. Higher scores correspond with higher amounts of symptoms.

Anxiety was assessed using the German Screening Test for Child Anxiety Related Emotional Disorders (SCARED; 64). The questionnaire’s reliability measure obtained from the printed test manual for each informant is: Cronbach’s alpha for mothers = 0.89, and for fathers = 0.93. The questionnaire is designed to assess children’s anxiety. It is composed of 41 items: 13 items about panic/somatic symptoms; nine items about generalized anxiety; eight items about separation anxiety; seven items about social phobia; and four items about school phobia. The reliability measures obtained from the printed test manual are: Cronbach’s alpha = 0.81, 0.81, 0.71, 0.75 and 0.66, respectively for the above-mentioned item groups. Items were scored on a four-point Likert scale (scores ranged between 0 and 3). The raw score (= summed score) of each scale is transferred to a standardized score. Higher scores correspond with higher amounts of symptoms.

Familial and environmental factors were assessed using a parental questionnaire. The questionnaire is designed to assess the parents’ familial and childhood background. Parents were presented with items about their: familial, own and children’s developmental problems and psychopathologies; experience with learning interventions; general familial history; level of obtained education; occupation; ethnicity; and lingual proficiencies. This variable was used as SES in the data analysis.

### Analyses

#### Data preparation

Data were prepared and analyzed using REDCap [65] and R software [66]. Data preparation included the transformation of raw scores to z-scores separated by grade. Standardization was based on grade-specific norms for academic and intelligence tests based on the current representative sample, and based on age and gender-specific norms provided by the test manual for the psychopathological questionnaires. For all variables, higher scores indicate higher levels of skills, symptoms or involvement. For psychopathologies, combined scores were calculated for internal and external disorders based on the mean of the standardized scores for anxiety and depression, and for ADHD and conduct disorder, respectively.

#### Planned data analyses

Data Analyses were performed in the R software[66] using the Lavaan package [67]. Maximum Likelihood was used as the estimator for all computed models. Moreover, for all models, criteria suggested by Hu and Bentler[68] were followed when evaluating model fit: χ^2^ not statistically significant (*p* > .050); comparative fit index (CFI) > 0.950; root mean square error of approximation (RMSEA) < 0.060; and standardized root mean square residual (SRMSR) < 0.080.

**Fig. 1:**
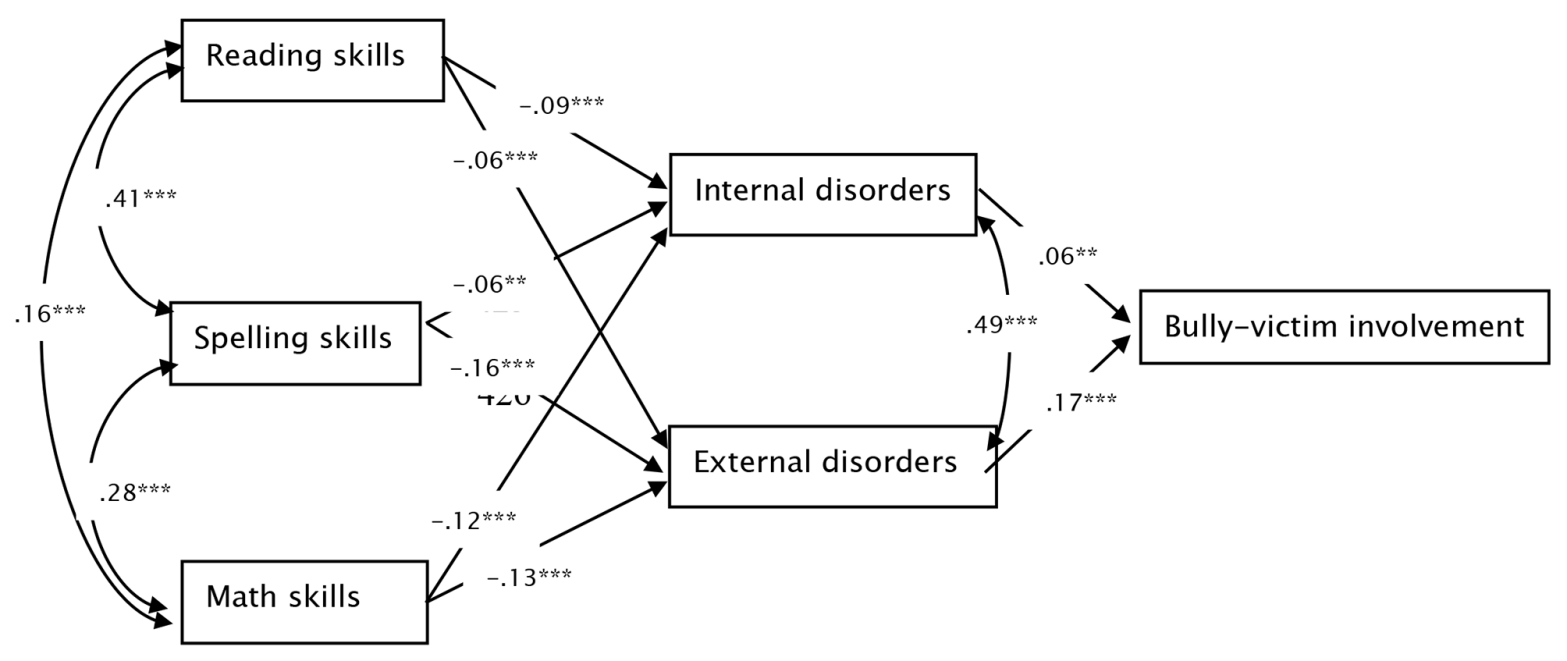
The final path model. *Note*: The reported values are standardized path coefficients. Significance values: *p* < .001 ‘***’, *p* < .01 ‘**’, *p* < .05 ‘*’

To answer research question [1] (Is there is a direct association between LD/learning skills and bullying, or does the association depend on other co-occurring disorders?), correlation analyses and path analyses were performed. To this aim, the first step was calculating correlations between the investigated variables. The second step was investigating all single paths by analyzing the following direct associations: is bully-victim involvement predicted by reading, spelling and math skills; is bully-victim involvement predicted by internal and external disorders; and are internal and external disorders predicted by reading, spelling and math skills. The third step was investigating two path models: the first included a direct path between LD and bullying; whereas the second did not (see Fig. 1). The two models were compared to test whether model fit is better with or without the direct path between LD and bullying.

To answer research question [2] (Do the associations differ when comparing children with and without LD?), a multigroup path analysis was performed. To this aim, a free model and a constrained model, with intercepts and path coefficients fixed to be identical for the two groups were compared. Then, as the models differed, the constraining of each path separately was explored. In order to clearly differentiate between children with and without LD, children scoring between − 1.5 SD and − 1 SD (assessments detailed above under “Children’s assessments”) were excluded from the analyses. Resultingly, the control group consisted of 2,180 of the children, and the LD group consisted of 373 children.

To answer research question [3] (Do these associations differ when examining either being both a bully and a victim compared to only being a victim or a bully), another path analysis was performed. To this aim, the variables described above were modeled with either only victimization or only bullying perpetration as the outcome variable. These models were compared to the model with combined bullying roles (i.e., bully-victim involvement) as the outcome variable to test whether models differ in either of the bullying roles.

To answer research question [4] (do the associations differ when examining different genders?), a multigroup analysis was performed (see multigroup path analysis description for research question 2). This analysis was performed to test whether there are gender differences in overall bullying involvement; in the overall model; and in any of the separate paths.

Finally, in order to assess whether the associations differ when taking IQ and SES into account, the variables described above were modeled with the addition of IQ and SES to the endogenous variables (internal and external disorders). This model was compared to the model without IQ and SES.

## Results

Group comparisons between children with and without LD across the study variables (Table 1) revealed that compared to children without LD, children with LD had worse reading, spelling and math skills and had higher levels of internalizing and externalizing symptoms. With respect to bully-victim involvement, there was a small significant group difference (when tested one sided based on previous research), suggesting that the LD group was more involved in bullying compared to children without LD. However, the effect size was relatively small.

**Table 1:** Mean differences of groups across study variables

Total N = 2553	No LD	SD	LD	SD	*p* - value	*Effect size (Cohen’s d)*
N	2180		373			
Reading	0.45	0.76	-1.04	1.03	< 0.001	1.85
Spelling	0.44	0.76	− 0.98	1.05	< 001	1.76
Math	0.37	0.72	− 0.78	1.05	< 001	1.47
Internalizing	− 0.13	0.94	0.29	1.01	< 001	− 0.44
Externalizing	− 0.18	0.95	0.41	1.01	< 001	− 0.61
Bully-victim inv.	− 0.04	0.83	0.04	0.87	0.086	− 0.10

*Note.* All variables indicate z-scores separated by grade. *SD* is used to represent standard deviation

Firstly, to answer research question [1] (Is there is a direct association between LD/learning skills and bullying, or does the association depend on other co-occurring disorders?), correlation analyses between the investigated variables were performed. Nearly all correlations were significant on a 0.01 level (Table 2), with the exception of the non-significant correlation between reading and bully-victim involvement. The correlations between bully-victim involvement, and learning skills (i.e., spelling and math skills) were all negative, indicating that poorer learning skills are associated with higher bully-victim involvement. The correlations between bully-victim involvement and both internal and external disorders were all positive and significant, indicating that more internalizing and externalizing symptoms are associated with higher bully-victim involvement. Notably, correlations seem to be higher for bully-victim involvement with externalizing disorders compared to with internalizing disorders. The correlations between all learning skills and internal and external disorders were negative and significant, indicating that poorer learning skills are associated with more internalizing and externalizing symptoms. Internalizing and externalizing disorders correlated similarly with learning skills.

**Table 2:** Means, standard deviations, and correlations with confidence intervals

N = 2,925	*M*	*SD*	1	2		3	4	5
1. Reading	0.17	0.98						
2. Spelling	0.16	0.96	0.45**					
			[0.42, 0.48]					
3. Math	0.11	0.90	0.20**	0.32**				
			[0.16, 0.23]	[0.29, 0.35]				
4. Internal disorders	-0.05	0.97	− 0.14**	− 0.14**		− 0.16**		
			[-0.18, − 0.10]	[-0.17, − 0.10]		[-0.19, − 0.12]		
5. External disorders	-0.07	0.98	− 0.17**	− 0.23**		− 0.18**	0.56**	
			[-0.20, − 0.13]	[-0.27, − 0.20]		[-0.22, − 0.15]	[0.53, 0.58]	
6. Bully-victim inv.	-0.02	0.83	− 0.03	− 0.07**		− 0.06**	0.19**	0.25**
			[-0.07, 0.00]	[-0.11, − 0.03]		[-0.10, − 0.03]	[0.15, 0.22]	[0.22, 0.29]

*Note. M* and *SD* are used to represent mean and standard deviation, respectively. Values in square brackets indicate the 95% confidence interval for each correlation. * indicates *p* < .050. ** indicates *p* < .010

Thereafter, three initial models were performed to test the direct associations between: bully-victim involvement and reading, spelling and math skills (*b* = 0.002, *t* = 1.30, *p* = .896; *b* = − 0.049, *t* = -2.658, *p* = .008; *b* = − 0.044, *t* = -2.412, *p* = .016, respectively); bully-victim involvement and internal and external disorders (*b* = 0.056, *t* = 3.007, *p* = .003; *b* = 0.183., *t* = 9.983 *p* < .001, respectively); and internal and external disorders and reading, spelling and math skills (*b* = − 0.089, *t* = -4.391 *p* < .001; *b* = − 0.058, *t* = -2.740, *p* = .006; *b* = − 0.133, *t* = -6.427, *p* < .001, respectively for internal disorders; *b* = − 0.073, *t* = -3.327 *p* < .001; *b* = − 0.166, *t* = -7.849, *p* < .001; *b* = − 0.127, *t* = -6.195, *p* < .001, respectively for external disorders). All three models were statistically significant (all *p* values < 0.001). This provided merit to continue with two more complex path models.

Specifically, reading, spelling and math skills were modeled as the exogenous variables. Internal and external disorders were modeled as the endogenous variables, and bully-victim involvement was modeled as the outcome variable. A model without the mediating path of internal and external disorders (i.e., the endogenous variables) showed that math and spelling skills were significant predictors of bully-victim involvement (*b* = − 0.040, *t* = -2.412, *p* = .016; *b* = − 0.049, *t* = -2.658, *p* = .008, respectively), while reading was not (*b* = 0.002, *t* = 0.130, *p* = .896). When adding the mediation to the model, none of the direct effects were significant, but internal and external disorders were significant predictors of bully-victim involvement (*b* = 0.056, *t* = 3.006, *p* = .003; *b* = 0.180, *t* = 9.646, *p* < .001, respectively). Moreover, for this model including the direct path, the following indices were obtained: χ^2^(0, *N =* 2925) < 0.001, *p* < .001; CFI = 1.00; RMSEA < 0.000; SRMSR < 0.000, which do not indicate a good fit to the data [68]. Next, as the direct paths between learning skills and bully-victim involvement were not statistically significant, a simpler model without the direct paths was analyzed. For this model, the following indices were obtained: χ^2^(3, *N =* 2925) = 2.34, *p* = .500; CFI > 0.990; RMSEA < 0.001; SRMSR = 0.005, which indicate a good fit to the data [68]. Even though a comparison between the models revealed that they do not differ significantly (*p* > .05), the second model was chosen as the final path model as it was simpler and fit the data better (Fig. 1).

Portions of model variance were explained by: internal disorders, *R*^2^ = 0.040 (4%); external disorders, *R*^2^ = 0.071 (7.1%); and bully-victim involvement, *R*^2^ = 0.066 (6.6%). Furthermore, the indirect effects of reading, spelling and math on the outcome variable (bully-victim involvement), through the endogenous variables (internal and external disorders) were all statistically significant (all *b* values between − 0.003 and − 0.030, and all *p* values between 0.001 and 0.043). These indirect effects were significant through internalizing disorders: reading (*ab* = − 0.005, 95% CI [-0.010, − 0.002]), spelling (*ab* = − 0.003, 95% CI [-0.010, − 0.001]), math (*ab* = − 0.007, 95% CI [-0.010, − 0.003]) and through externalizing disorders: reading = *ab* = − 0.010, 95% CI [-0.020, − 0.010], spelling = *ab* = − 0.030, 95% CI [-0.040, − 0.020] and math = *ab* = − 0.020, 95% CI [-0.030, − 0.020]).

Next, to answer research question [2] (Do the associations differ when comparing children with and without LD?), a multigroup path model with the two groups (LD and a control group) was performed (see Table 3 for the correlations for each group). The explained variance values in the LD group were for the most part slightly higher than those observed in the control group: internal disorders, *R*^2^ = 0.036 (3.6%) vs. *R*^2^ = 0.013 (1.3%), external disorders, *R*^2^ = 0.077 (7.7%) vs. *R*^2^ = 0.027 (2.7%), and bully-victim involvement, *R*^2^ = 0.054 (5.4%) vs. *R*^2^ = 0.068 (6.8%). The multigroup path model was respecified in two group variations: a free model and a constrained model, with intercepts and path coefficients fixed to be identical for each of the groups. For these models, the following indices were obtained: χ^2^[6] = 3.398, *p* = .758; CFI > 0.990; RMSEA < 0.001; SRMSR = 0.007; χ^2^[20] = 765.316, *p* < .001; CFI = 0.492; RMSEA = 0.171; SRMSR = 0.153, respectively. These indices indicate that the free model fits the data better than the constrained model [68]. A comparison between these models revealed that the difference between the free and the constrained model was statistically significant (*p* < .001), indicating that the models for the two groups are not identical. In order to identify if specific paths differ between groups, we released the constraints of each path one-by-one. This analysis revealed that the groups differed only in the path between spelling and externalizing symptoms. However, for both groups, the path was highly significant (*p* < .001) albeit slightly more pronounced in the LD group (*b* = − 0.237, *t* = -4.925, *p* < .001; *b* = − 0.127, *t* = -4.479, *p* < .001, respectively for the LD group and the control group).

**Table 3:** Correlations for the LD group and the control group with confidence intervals

LD	1	2	3	4	5
1. Reading					
2. Spelling	0.15**				
	[0.05, 0.25]				
3. Math	− 0.34**	− 0.11*			
	[-0.43, − 0.25]	[-0.21, − 0.01]			
4. Internal disorders	− 0.04	− 0.12*	− 0.12*		
	[-0.14, 0.06]	[-0.21, − 0.01]	[-0.22, − 0.02]		
5. External disorders	0.01	− 0.23**	− 0.13*	0.60**	
	[-0.09, 0.11]	[-0.33, − 0.13]	[-0.22, − 0.02]	[0.54, 0.67]	
6. Bully-victim inv.	0.03	− 0.09	− 0.08	0.12*	0.23**
	[-0.07, 0.13]	[-0.19, 0.02]	[-0.18, 0.02]	[0.02, 0.22]	[0.13, 0.33]
Control					
1. Reading					
2. Spelling	0.29**				
	[0.26, 0.33]				
3. Math	0.08**	0.21**			
	[0.04, 0.13]	[0.17, 0.25]			
4. Internal disorders	− 0.07**	− 0.06**	− 0.09**		
	[-0.11, − 0.03]	[-0.10, − 0.02]	[-0.13, − 0.05]		
5. External disorders	− 0.09**	− 0.13**	− 0.11**	0.52**	
	[-0.13, − 0.05]	[-0.17, − 0.09]	[-0.15, − 0.07]	[0.49, 0.55]	
6. Bully-victim	− 0.03	− 0.06**	− 0.05*	0.18**	0.25**
	[-0.07, 0.01]	[-0.10, − 0.01]	[-0.09, − 0.01]	[0.14, 0.22]	[0.21, 0.29]

*Note.* Values in square brackets indicate the 95% confidence interval for each correlation. * indicates *p* < .050. ** indicates *p* < .010

After that, to answer research question [3] (Do these associations differ when examining either being both a bully and a victim compared to only being a victim or a bully), the outcome variable for the final model described above, was respecified. Specifications were to either have only victimization or only bullying perpetration as the outcome variable. Again, the pattern observed when the outcome variable was only victimization or only bullying perpetration was comparable to the one observed with the combined bully-victim involvement variable: internal disorders, *R*^2^ = 0.040 (4%) vs. *R*^2^ = 0.040 (4%) vs. *R*^2^ = 0.040 (4%); external disorders, *R*^2^ = 0.058 (5.8%) vs. *R*^2^ = 0.071 (7.1%) vs. *R*^2^ = 0.071 (7.1%); and victim/bully/bully-victim involvement, *R*^2^ = 0.058 (5.8%) vs. *R*^2^ = 0.039 (3.9%) vs. *R*^2^ = 0.066 (6.6%).

Afterwards, to answer research question [4] (do the associations differ when examining different genders?), an independent t-test was used to examine potential gender differences in bully-victim involvement. There was a significant difference in bully-victim involvement between boys (*M* = 0.049, *SD* = 0.850) and girls (*M* = − 0.099, *SD* = 0.810), wherein boys demonstrated significantly higher bully-victim involvement, *t*(2921) = 4.82, *p* < .001. Thereafter, a multigroup path model was respecified in two gender variations: a free model and a constrained model, with intercepts and path coefficients fixed to be identical for each of the genders. For these models, the following indices were obtained: χ^2^[6] = 8.322, *p* = .215; CFI = 0.999; RMSEA = 0.016; SRMSR = 0.010; χ^2^[20] = 133.801, *p* < .001; CFI = 0.955; RMSEA = 0.062; SRMSR = 0.040, respectively. These indices indicate that the free model fits the data better than the constrained model [68]. A comparison between these models revealed that the difference between the free and the constrained model was statistically significant (*p* < .001), indicating that the models are not identical for males and females. Next, a series of models was used to explore each path separately by releasing the constraints of each path one-by-one. This analysis revealed that the difference between males and females was not statistically significant in any of the single paths.

Finally, to assess whether associations differ when taking IQ and SES into account, the endogenous variables for the final model described above, were respecified. Due to missing items, the resulting sample size decreased to *N =* 2454. Interestingly, only IQ accounted for a significant amount of variance of the outcome variable. For the most part, in comparison with the final model described above, there was a decrease in the portions of model variance explained by, respectively: internal disorders, *R*^2^ = 0.028 (2.8%) vs. *R*^2^ = 0.040 (4%); external disorders, *R*^2^ = 0.060 (6%) vs. *R*^2^ = 0.071 (7.1%); and bully-victim involvement, *R*^2^ = 0.072 (7.2%) vs. *R*^2^ = 0.066 (6.6%).

## Discussion

Both LD and bullying are major sources of public concern [6, 20]. Previous research on the interplay between the two yielded inconsistent patterns. Specifically, it was not clear whether LD are a direct childhood bullying risk factor, or whether the association depends on co-occurring disorders. The current study is the first to demonstrate that LD are not a direct childhood bullying risk factor. Rather, LD are only a risk factor when there are co-occurring psychiatric symptoms. Namely, once children have LD, they are more likely to also suffer from psychiatric comorbidity, and consequently their risk of being involved in bullying as both bullies and victims increases (Fig. 1). These findings suggest that for such children, early bullying prevention could be useful to hinder consequential negative effects.

The current study also sought to investigate sample and bullying role differences, as well as other potentially relevant factors, namely gender, IQ, and SES.

In terms of sample differences, comparing the models between children with and without LD revealed an overall difference between the two groups. However, when analyzing each path separately, significant differences were only found in the path between spelling and externalizing disorders, wherein a negative effect size was more pronounced in the LD group, even though the path was highly significant for both groups.

In terms of different bullying roles, we did not find differences between models. One possible reason is that children with LD involved in bullying are prone to be both victims and bullies. This is in line with previous research arguing for bully-victim duality (e.g., 51). This duality could be explained as follows: children with LD often find it harder to socialize with their peers (e.g., 18); as a result, they have less protection from their social group, and are at greater risk of being rejected and bullied [17]; some victims react aggressively to bullying, and thus, in turn, they become bullies as well [51]; in parallel, children with LD are likely to suffer from co-occurring disorders (e.g., ADHD; 14); such co-occurrences, especially co-occurrences with externalizing disorders, increase the likelihood of victims reacting aggressively to bullying, and thus, the bully-victim duality is reinforced [52].

In terms of gender, gender differences were not found in any of the specific paths of the final model (Fig. 1). Nonetheless, an overall difference did emerge, wherein boys were more involved in bullying compared to girls. This gender difference is compatible with some past research investigating the role of LD as childhood bullying risk factors (e.g., 40, 43, 46). In terms of IQ and SES, it is likely that little to no differences in the models were found when they were controlled, because involvement in bullying is influenced by many factors. Moreover, although IQ accounted for a significant amount of variance in the outcome variable, including IQ did not improve model fit, likely due to an overlap with learning skills. This is in contrast to previous research that has found that IQ and SES are prominent bullying risk factors (e.g., 34). It is possible that this discrepancy is due to IQ and SES being indeed risk factors, but lessening in influence when examined alongside other factors. Alternatively, participants could have belonged to the higher end of the SES distribution (i.e., the sample has less variation). It is possible that the predictive patterns are different in the higher end of the distribution compared to the full range of SES. In terms of future research, it would be useful to ensure the sample encompasses the full range of SES.

Several limitations merit a brief discussion. Firstly, the total explained variance of bullying in the current study was only 6.6%. Similarly, for Klomek et al. [43], the total explained variance of victimization was 7%. They rationalized this by stating that there are numerous other potentially influential factors that could be accounted for in future research. Nevertheless, their study was the first to examine the association between LD, ADHD and bullying, and contributed to the development of the research field. The current study adds to the existing literature by examining the association between different LD (i.e., reading, spelling and math disorders), several comorbid disorders and different bullying roles. Although the current study could not control for all potentially relevant environmental factors (e.g., social support), it did control for gender, IQ and SES, factors that did not increase the explained variance. Secondly, the current study had a cross-sectional design, and participants’ mean age was 9.72 years, whereas bullying is most prominent at ages 11–13 [69]. In terms of future research, it would be useful to adapt the model to a longitudinal design to better understand the developmental trajectories, and to also sample older children. Thirdly, bullying involvement was measured using self-report only, and did not account for the social context in which bullying naturally occurs. Using such a measurement could have led to an underrepresentation, as discussed by Fogler et al. [70], especially since bullying is often considered a socially undesirable behavior. In terms of future research, different measurements (e.g., peer popularity ratings) should be used in order to incorporate the social context and encourage a more realistic representation. Despite these limitations, the current study enhances the understanding of the link between comorbid LD and bullying, and will hopefully stimulate further investigation of this important area.

The study has the following practical implication: First, it confirms that bullying is another hurdle children with comorbid LD might have to face. Second, it provides merit for school professionals to pay more attention to children with comorbid LD, and enforce pre-defined policies on how to respond to acts of bullying towards and among these children. Third, it also provides merit to focus on children with comorbid LD as a specific risk group in bullying intervention programs. As the association found between LD and bullying is compatible with the assumption of bullying role duality (i.e., being both a victim and bully), such interventions should encompass coping strategies for both roles. In addition to program inclusion, personal support could be offered to children with comorbid LD that have been recognized by parents or school professionals as prone to being involved in bullying. Finally, the association found in the current study could serve as theoretical grounding for future research investigating the association between comorbid LD and cyberbullying. Amid the Covid-19 pandemic, as more traditional schooling was replaced with online learning, alongside traditional bullying, cyberbullying has emerged as an issue of utmost relevance [71]. Moreover, the daily amount of time youths spend on a computer increases the risk of cyberbullying involvement [72]. Using interactive computer software, such as Cyberball [73], cyberbullying could be experimentally simulated (e.g., 74 used Cyberball to simulate cyberbullying in participants with depression and borederline personality disorder via a virtual ball tossing game with other participants). Future research could compare Cyberball responses of children with comorbid LD with those of typically developing controls. In the domain of LD, a few studies have already used self-report and parental questionnaires to show that comorbid LD increases the likelihood of cyberbullying involvement (e.g., 75, 76). Future research could contribute to this important research realm by comparing traditional bullying to cyberbullying among children with and without comorbid LD, incorporating interactive simulations, and taking into account further factors such as social support and the daily amount of time children spend online.

## Conclusion

The current study demonstrated that learning disorders are an indirect childhood bullying risk factor, as their impact depends on psychiatric comorbidity with internalizing (i.e., anxiety and depression) and externalizing disorders (i.e., ADHD and conduct disorder). The importance of this finding is the identification of children with such co-occurrences as a risk group, to which anti bullying interventions could be tailored and enforced. Furthermore, this finding adds to previous inconsistent findings on the association between LD and bullying by showing that there is an indirect rather than a direct relationship. Moreover, the study provides grounds for future studies with this risk group (i.e., LD with psychiatric comorbidity), encompassing both traditional bullying and cyberbullying, with the latter becoming increasingly widespread as many children increased their online interactions amid the Covid-19 pandemic.

## Data Availability

The datasets used and/or analyzed during the current study are not publicly available due to further planned research, but are available from the corresponding author on reasonable request.

## Abbreviations

ADHD: Attention-deficit/hyperactivity disorder
BMI: Body mass index
CODY-M 2–4: Cody math test
CFI: comparative fit index
CFT 20-R: Culture Fair Intelligence Tests
DISYPS-II: Psychopathological profile questionnaire
DSM-5: The Diagnostic and Statistical Manual of Mental Disorders, Fifth Edition
LD: Learning disorders
LGBTQ+: Lesbian, gay, bisexual, transgender and related communities
OBQ: Olweus bully/victim questionnaire
RMSEA: Root mean square error of approximation
SCARED: Screening Test for Child Anxiety Related Emotional Disorders
SD: Standart deviations
SES: Socioeconomic status
SRMSR: Standardized root mean square residual
WLLP-R: Würzburger silent reading test – revised
WRT 3+: Weingartener spelling test for basic vocabulary for 3rd graders
WRT 4+: Weingartener spelling test for basic vocabulary for 4th graders
